# Proximate composition, phytochemicals, minerals and antioxidant activities of Vigna mungo L.seed coat

**DOI:** 10.6026/97320630015579

**Published:** 2019-09-10

**Authors:** Pushpam Marie Arockianathan, Kumar Rajalakshmi, Priya Nagappan

**Affiliations:** 1 PG and Research Department of Biochemistry, St. Joseph's College of Arts and Science (Autonomous), Cuddalore-607001, Tamil Nadu, India

**Keywords:** Vigna mungo L, antioxidant, proximate analysis, phytochemical analysis, seed coat

## Abstract

Vigna mungo L. seed coat is an agro by-product, available in substantial quantity, with no clear evidence to prove its economic importance. In the present study, the phytochemicals, proximate content, minerals and in vitro antioxidant activities of aqueous (AE), ethanol (EE) and 80% ethanolic extract (80% EE) of Vigna mungo L. seed coat were determined. The total carbohydrate, total protein, crude fibre, ash and moisture content of the seed coat were found to be 27.52±2.71%, 10.07±0.92%, 48.67±1.96%, 4.87±0.29% and 11.03±0.49% respectively. The content of total phenolics and flavonoids were significantly higher in 80% EE than other extracts. The mineral composition showed that seed coat was rich in calcium, sodium, potassium, magnesium, iron, copper, zinc and manganese. The higher antioxidant potential was shown by 80% EE in DPPH and SOD assay whereas AE shows more scavenging activity in H2O2 assay. So it can be used as neutraceuticals in food supplements.

## Background

Legumes are widely grown throughout the world and its dietary importance is appreciated globally. Legumes serve as supplementary proteins for a large human population and also add variety of nutrients to the diet. They are valuable sources of complex carbohydrates, proteins and dietary fibre which contribute significant amounts of vitamins, minerals and have high energy value [1]. Vigna mungo L. is also known as mash bean which belongs to the family Leguminosae. India is the major producer and consumer of black gram with the production of 1.82 million tons annually. This can be grown under low moisture and fertility conditions. Black gram (Vigna mungo L.) is a major important pulse cultivated not only in India, but also in other Asian countries and some parts of Africa. The name 'Black gram' was given to it due to the colour of its seed coat.The chicken seekh kababs from meat of spent hens can be successfully extended with black bean [2]. Lipid content in black gram was shown to reduce cholesterol in both humans and experimental animals [3]. The seed coat of cereals and legumes have large quantities of endogenous antioxidants such as phenolic compounds [4,5]. However the use of flours as ingredients in food processing is dependent on its functional properties. The functional properties directly or indirectly affect the processing applications and food quality. It is also reported that legumes have certain phytochemicals like polyphenols, flavonoids, phytosterols that provide various health benefits [6,7,8]. Large quantities of pulse husks are available as by-product and are available to the extent of 3 million tonnes in India per annum [9]. The principle objective of supplementation is to increase the supply of nutrients, mainly energy and protein, which enhance the basal roughage in rumen [10].

Vigna mungo seed coat protects the seed not only from mechanical stress but also from pathogens invasion and also from temperature, humidity fluctuations during storage. Phenolic compounds in the seed coat contribute to seed hardness and inhibitions of microbial growth. During germination the seed coat protects the seed from hydration stress and electrolyte leakage. However, very little information is available in the literature about Vigna mungo seed coat. In the present study, the seed coat of Vigna mungo L. was assessed for its proximate composition, phytochemicals, minerals and antioxidant potential in order to bring out its economic and health benefits.

## Materials and methods

### Materials

Tannic acid, 2,2-diphenyl-1-picryhyrazyl (DPPH), ascorbic acid (ASA) were purchased from Sigma Aldrich Chemical Co. (St.Louis, USA). Folin-Ciocalteu phenol reagent was obtained from SRL Limited. All other chemicals and solvents used in this study were of analytical grade.

### Preparation and extraction of samples

Vigna mungo seeds were procured from local market Cuddalore, Tamil Nadu, India.The seed coat was separated from Vigna mungo seeds and shade dried at room temperature. The dried seed coat material was powdered mechanically using commercial electrical stainless steel blender. Different extracts (aqueous (AE), ethanol (EE) and 80% ethanol (80%EE)) were prepared using Vigna mungo seed coat using the following protocol. 100% ethanolic extract was less efficient to extract low molecular weight phenolic compounds with high antioxidant capacity from the extract as compared to 80% ethanolic extract. So, 80% ethanolic extract was used to assess its potential apart from pure ethanolic and aqueous extract. 20 gms of dry Vigna mungo seed coat powder were prepared and transferring into 200 mL of water and kept it on the magnetic stirrer for 24 hrs, these mixture were filtered through Whatman No.1 filter paper. The filtrate was then dried in incubator at 40 °C until the sample dried. Now, the dried extract was used for further analysis.

### Phytochemical studies

The phytochemical screening of different extracts of Vigna Mungo L. seed coat was carried out according to standard methods for the following chemical compounds such as alkaloids, terpeneoids, phenols, tannins, carbohydrates, saponins, flavonoids, proteins and sterols [11].

### Determination of total phenolic content

The total phenolic content of the extract was determined by Folin- Ciocalteu method [12]. An aliquot of 1 mL of extract was mixed with 5 mL of Folin-Ciocalteau reagent and sodium carbonate solution. The mixture was allowed to stand for 40 min in dark and the absorbance was read at 764 nm. The results were expressed on a dry matter basis. The total phenolic content was calculated from tannic acid standard curve.

### Determination of total flavonoid content

The total flavonoid content of the extract was determined by Zhishen et al method [13]. An aliquot of 1 mL extract was made up to 3 ml with distilled water, add 0.03ml of Sodium nitrate solution and incubated for 5 min at 25°C followed by the addition of 0.03ml of 10%AlCl3 and the mixture was allowed to stand for 5min. Finally the reaction mixture was treated with 0.2ml of 1mM NaOH and the absorbance was read at λ = 510 nm. The flavonoid content was calculated from a quercetin standard curve.

### Proximate analysis

The proximate composition of Vigna mungo L. seed coat was determined using standard methods [12]; for moisture content drying at 105 °C; for ash content in muffle furnace at 550 °C; Crude fat by solvent extractor apparatus with petroleum ether as solvent; and crude protein by Lowry et al method [14]. Total carbohydrate was estimated by difference method. All analysis was carried out in triplicate.

### Mineral analysis

The mineral composition of Vigna mungo L. seed coat was determined using Thermo scientific Atomic absorption spectrophotometer (Model iCE^TM^3000). 2 gm of sample was heated in a muffle furnace to get the ash. The resulting ash was solubilised using nitric acid and hydrochloric acid in the ratio 1:3 then, the supernatant solution obtained was used for the determination of mineral content. The values were expressed on dry matter basis.

### Antioxidant activity

DPPH radical scavenging activity

DPPH radical scavenging activity of the sample was determined by 1,1-diphenyl-2-picryl-hydrazyl assay [15]. An aliquot of 0.5 mL of test sample solution (25,50 and 100 µg/mL) in methanol was mixed with 2.5 mL of 0.5 mM methanolic solution of DPPH. The mixture was shaken vigorously and incubated for 30 min in dark at room temperature. The absorbance was measured at 517 nm using UV spectrophotometer against standard ascorbic acid. The % inhibition was calculated using the formula

% inhibition = absorbance of control-absorbance of sample/ absorbance of control

### SOD activity

SOD activity was measured by the method of Liu et al. [16]. The reaction mixture consists of 1 mL of 50 µM nitroblue tetrazolium (NBT) and 1 mL of 78 µM NADH in 3 mL of 16 mM Tris-HCl buffer mixed with sample extract ( 25-100 µg/mL) in water. Then, 1 mL of 10 M phenazine methosulfate added to this mixture which was incubated at 25 °C for 5 min. The reaction was started adding 1 mL of 10 M PMS to the mixture. The reaction mixture was incubated at 25°C for 5 min.The colour developed was absorbed and recorded at 560 nm using standard ascorbic acid. The % inhibition of superoxide ion was calculated using the formula

% inhibition = absorbance of control-absorbance of sample/ absorbance of control

### Estimation of hydrogen peroxide

Hydrogen peroxide was determined by the method of Ruch et al [17]. A solution of hydrogen peroxide (4mM) was prepared in phosphate buffer (pH 7.4).The sample concentration of 10-100 µg/ mL was added to 0.6 mL 40mM hydrogen peroxide then the mixture was measured at 230 nm after 10 min against blank solution without hydrogen peroxide in phosphate buffer solution.The content was expressed as µmol/g. The % inhibition of hydrogen peroxide was calculated using the formula % inhibition = absorbance of control-absorbance of sample/ absorbance of control

### Statistical Analysis

Data are expressed as mean ± SD. Values are compared using Duncan's multiple comparison using SPSS 13 software. P values less than 0.05 were considered statistically significant.

## Results and Discussion

Vigna mungo L seed coat is an important feed for livestock in some regions of India [18].The importance of the seed coat has not been explored fully in terms of nutrition, mineral and antioxidant potential.

## Phytochemical screening

In this study, the seed coat of Vigna mungo L. showed appreciable amount of alkaloids, glycosides, flavonoids, terpenoids, phenols and tannins (Table 1). Among the phytochemicals, the presences of phenolic and flavonoid compounds are prominent. Phenolic compounds have the therapeutic property on different diseases like diabetes, asthma, allergy, cancer, bacterial, viral infections etc. Flavonoids possess a broad spectrum of chemical and biological activity including free-radical scavenging property [19].

## Total phenolics and flavonoids content

**Figure 1:**
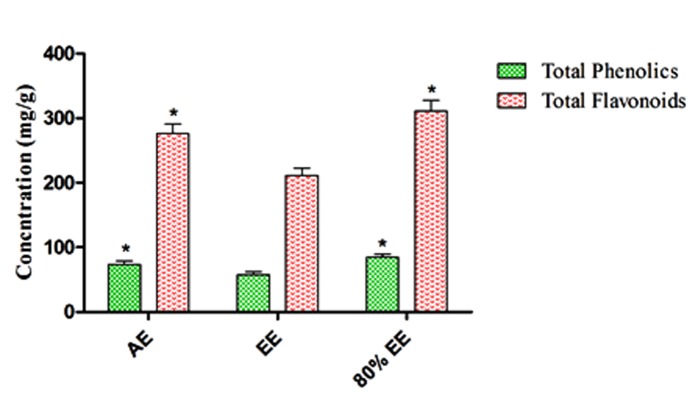
The total phenolic and flavonoid content of different extracts (AE-Aqueous, EE-Ethanol and 80% EE-80% Ethanolic extract) of Vigna mungo L. seed coat. The concentration of phenolics and flavonoids were expressed as mg tannic acid (TAE) equivalent/g and mg quercetin equivalent/g respectively. Results are presented as Mean ± SD; statistically significant data are given as p < 0.05.

The hydroxyl groups present in phenolics facilitate the free radical scavenging ability of the compounds. So the determination of total phenolic concentration in samples form the basis for quick screening of antioxidant potential of plants. The total phenolics levels determined in this way are not absolute measurements of the amounts of phenolic compounds, but are in fact based on their chemical reducing capacity relative to tannic acid (TAE) as standard. Total phenolics in 80% ethanolic extract was 85.45± 5.1 mg/g tannic acid equivalents (TAE) while the aqueous and ethanolic extract were 73.55 ±5.3 mg/g (TAE) and 57.78 ±3.1 mg/g (TAE) respectively (Figure 1). These results indicate that 80% ethanolic extract show more potential effect of phenolics than other extracts. The high phenolic contents in 80% ethanolic extract contribute to higher antioxidant activity.

Flavonoids occur naturally in plants, fruits and food products. It effectively scavenges most free radicals including singlet oxygen [20]. It exhibits vast pharmacological and therapeutic properties like antiviral, anti-inflammatory, anticancer, anti-diabetic and antiallergic activities [21,22,23]. Flavonoids act as natural antioxidants by suppressing reactive oxygen formation, chelating metal elements involved in free-radical production, scavenge free radicals and also promote antioxidant defences [24]. The total flavonoid content of aqueous, ethanolic and 80% ethanolic extracts were 256±11.65, 211±9.38 and 311±10.75 mg/g quercetin equivalents respectively (Figure 1). The 80% ethanolic extract exhibit significantly higher flavonoid content compared to other extracts.

## Proximate composition

The proximate composition of Vigna mungo L. seed coat was shown in Table 2. The moisture content of the seed coat was found to be 11.03 ± 0.49%. As moisture content is low, this sample is stable and can be stored. The total protein and fat content of the seed coat were found to be 10.075 ± 0.920 % and 0.46±0.121 % respectively (Table 2). Generally, ash content is measured for the quality assessment and for the functional properties of foods [25]. So the seed coat contains appreciable amount of total ash, which indicates high content of minerals. The dietary fibre plays an important role in our diet composition as it helps in preventing many diseases like diabetes, constipation, cardio vascular disease etc [26]. The result showed the seed coat contains rich amount of crude fibre. Many plants possess variety of phytochemical, which is associated with many pharmacological and therapeutic applications [27,28].

## Mineral composition

The mineral content present in seed coat were shown in Table 3. The minerals present in seed coat were determined based on dry weight basis. The mineral calcium was found to be highest and followed by sodium, magnesium and potassium. The seed coat also showed the appreciable amounts of iron, copper, zinc and manganese. The calcium is present in abundant quantity in the seed coat. So it can be considered an important dietary supplement to maintain the biological role of nerve transmission, contraction of muscles and also helps in mediated vasodialation and contraction [29]. This element also facilitates the efficient release of insulin from beta cells [30]. The dietary supplementation of calcium plays a pivotal role in lowering serum cholesterol level [31]. Due to this, the seed coat shows hypolipidemic properties as it has rich content of calcium. Potassium, the principle intracellular cation, regulates both pH and osmotic pressure. Copper is an important co-factor of enzymes involved in iron metabolism and also required for many protein functions like SOD, cytochrome C-oxidase, tyrosinase etc [32]. Zinc and manganese also play a key role in various metabolic pathways. As zinc is an integral part of many key enzymes, including DNA synthesis and repair, its deficiency leads to many diseases. So, from this study, the mineral composition of seed coat of Vigna mungo L. showed the presence of key trace elements, which is vital for our metabolic functions.

## Antioxidant potential

DPPH radical scavenging activity

**Figure 2:**
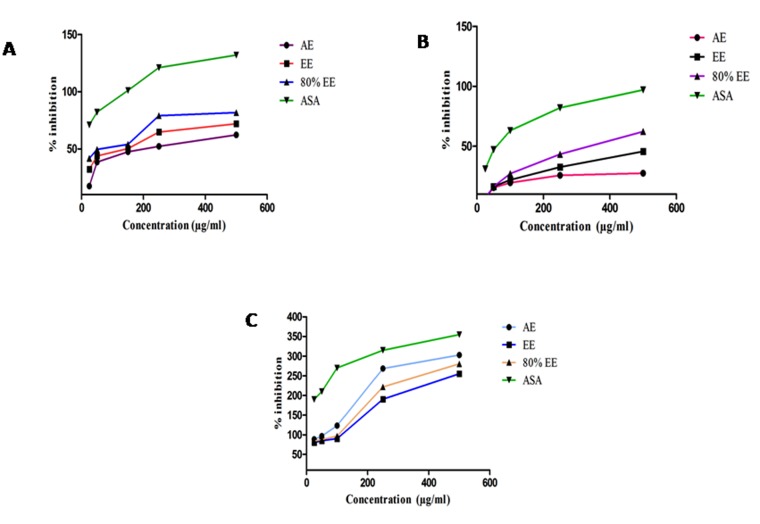
(A) DPPH scavenging activity; (B) SOD scavenging activity; (C) H_2_O_2_ scavenging activity of different extracts (AEAqueous, EE-Ethanolic and 80% EE-80% Ethanolic extract) of Vigna mungo L.seed coat with reference to Ascorbic acid (ASA) as standard.

DPPH is a stable free radical which is used to determine the free radical scavenging abilities of antioxidants present in plant extracts [33,34]. The antioxidant activity is then measured by the decrease in absorption at 515 nm by proton scavengers. [Figure 2A] illustrates the scavenging ability of different extracts. The DPPH scavenging activity of aqueous, ethanolic and 80% ethanolic extracts were found to be 62.216 ± 0.28%, 71.9 ± 0.99 % and 81.718 ± 0.43 % respectively at a concentration of 500 µg/mL (Figure 2A). The absorbance of DPPH was more rapidly decreased at 517 nm in the presence of 80 % EE followed by EE and then AE at an IC50 concentration of 49.75 and 245 µg/mL respectively (Figure 2A). This indicates that 80% EE possess more antioxidant activity in terms of hydrogen atom donating capacity. The reduced levels of hydrogen donors may be the reason for decreased level of free radical scavenging abilities of ethanolic and aqueous extract.

## Superoxide scavenging activity

Superoxide is a weak oxidant which gives rise to dangerous hydroxyl and singlet oxygen radicals. This leads to oxidative stress [35]. SOD radical is the main source of reactive oxygen species [36]. The SOD scavenging ability of different extracts was presented in (Figure 2B). In this assay, the plant extracts inhibit the formazon formation by NBT oxidation. The SOD scavenging activity of aqueous, ethanolic and 80% ethanolic extracts were found to be 27.495 ± 0.13%, 45.619 ± 0.28 % and 62.21 ± 0.18 % respectively at a concentration of 500 µg/mL (Figure 2B).

## Hydrogen peroxide radical scavenging activity

Hydrogen peroxide, a weak oxidising agent, can cross cell membrane and inactivate few enzymes by oxidation of its -SH group. It also produces many toxic effects when it reacts with Fe2+ and Cu2+ ions to form powerful oxidising hydroxyl radicals. The phenolic compounds act both as hydrogen donors and hydrogen acceptors, thereby scavenges free radicals [37]. The H_2_O_2_ scavenging ability of different extracts was presented in Table 9. The H2O2 scavenging activity of aqueous, ethanolic and 80% ethanolic extracts were found to be 302.84 ± 1.68 %, 255.37 ± 1.84% and 280.46 ± 1.28% respectively at a concentration of 500 µg/mL (Figure 2C). H_2_O_2_ is not reactive at low concentration and it is rapidly decomposed to form hydroxyl radicals, which are cytotoxic in nature. So, it should be eliminated to protect the cell from damage [38].

## Conclusion

Vigna mungo L. (Black gram) is used widely as food products but their by-products mainly seed coat are thrown as waste or used as animal feed. The seed coat of Vigna mungo are found to be rich in flavonoids, glycosides, alkaloids, phenolics, carbohydrate and a group of polyphenolic antioxidants that are apparently responsible for free radicals scavenging effects. They are rich in fibre and protein. It also possesses important trace elements like calcium, sodium, potassium, magnesium, manganese, iron, copper, zinc etc. So the seed coat of Vigna mungo not only exhibit good antioxidant properties but also rich in phytochemicals, minerals, protein and fibre. Thus, the economic importance of Vigna mungo seed coat, an agro industrial by-product, can be explored for future use in food supplements and also for other health benefits.

## Figures and Tables

**Table 1:** Phytochemical Screening Vigna mungo L. seed coat

S. No	Phyto chemical Tests	Aqueous Extract	Ethanol Extract	80% Ethanol Extract
1	Alkaloids test	+	+	+
2	Glycosides test	+	+	+
3	Saponins	-	-	-
4	Oil test	-	-	-
5	Phenolic and tannins	+	+	++
6	Lead acetate	+	+	+
7	Gum test	-	-	-
8	Terpenoids:	-	-	+
9	Carbohydrate			
	a. Molisch's test	+	+	+
	b. Fehling's test	+	+	+
	c. Benedict's test	+	+	+
10	Diterpenes	+	+	+
11	Flavonoid test	+	+	++
+ present, - absent

**Table 2:** Proximate analysis of Vigna mungo L. seed coat

Quantitative Analysis test	Results (%)
Reducing sugar(in carbohydrate)	97.333 ± 8.2
Total carbohydrate	27.520± 2.7
Total protein	10.075 ±0.9
Moisture content	11.034±0.5
Total fat	0.46 ± 0.1
Crude fibre	48.67 ±2.0
Ash content (Dry Basis)	4.87 ± 0.3
Data are expressed in Mean ±SD (n=3)

**Table 3:** Mineral content (mg / 100g dry weight) of Vigna mungo L. seed coat

S .No	Minerals test	mg / 100g
1	Calcium	1062.85 ± 17.48
2	Sodium	523.47 ± 15.11
3	Magnesium	440.41± 13.80
4	Potassium	304.02± 3.58
5	Ferrous	10.47± 1.75
6	Manganese	6.38± 0.87
7	Copper	1.57± 0.05
8	Zinc	1.15± 0.04
9	Selenium	-Nil-
Data are expressed in Mean± SD (n=3)
